# Partial Deletion of Chromosome 8 β-defensin Cluster Confers Sperm Dysfunction and Infertility in Male Mice

**DOI:** 10.1371/journal.pgen.1003826

**Published:** 2013-10-24

**Authors:** Yu S. Zhou, Sheila Webb, Laura Lettice, Steve Tardif, Fiona Kilanowski, Christine Tyrrell, Heather MacPherson, Fiona Semple, Peter Tennant, Tina Baker, Alan Hart, Paul Devenney, Paul Perry, Tracey Davey, Perdita Barran, Chris L. Barratt, Julia R. Dorin

**Affiliations:** 1 MRC Human Genetics Unit, MRC IGMM, University of Edinburgh, Western General Hospital, Edinburgh, Scotland, United Kingdom; 2 Reproductive and Developmental Biology, Medical School, University of Dundee, Ninewells Hospital, Dundee, Scotland, United Kingdom; 3 EM Research Services, Newcastle Medical School, Newcastle University, Newcastle, England, United Kingdom; 4 School of Chemistry, Joseph Black Building, Edinburgh, Scotland; Stanford University School of Medicine, United States of America

## Abstract

β-defensin peptides are a family of antimicrobial peptides present at mucosal surfaces, with the main site of expression under normal conditions in the male reproductive tract. Although they kill microbes *in vitro* and interact with immune cells, the precise role of these genes *in vivo* remains uncertain. We show here that homozygous deletion of a cluster of nine β-defensin genes (*DefbΔ9*) in the mouse results in male sterility. The sperm derived from the mutants have reduced motility and increased fragility. Epididymal sperm isolated from the cauda should require capacitation to induce the acrosome reaction but sperm from the mutants demonstrate precocious capacitation and increased spontaneous acrosome reaction compared to wild-types but have reduced ability to bind the zona pellucida of oocytes. Ultrastructural examination reveals a defect in microtubule structure of the axoneme with increased disintegration in mutant derived sperm present in the epididymis cauda region, but not in caput region or testes. Consistent with premature acrosome reaction, sperm from mutant animals have significantly increased intracellular calcium content. Thus we demonstrate *in vivo* that β-defensins are essential for successful sperm maturation, and their disruption leads to alteration in intracellular calcium, inappropriate spontaneous acrosome reaction and profound male infertility.

## Author Summary

β-defensins are small molecules, considered primarily to be antimicrobials and important in the first defence response to invading organisms. They are predominantly produced at surfaces in contact with the outside environment and these include skin, airway and reproductive tract. We show here that when we delete from the mouse a subset of nine β-defensin genes, surprisingly the main consequence is that the male mice are completely infertile. When normal sperm leave the male and enter the female reproductive tract they are triggered to undergo a reaction that alters the membrane properties of the sperm and allows fertilisation. We show here that sperm isolated from the male mice, that no longer make these β-defensins, are prematurely ready to fertilise an egg. It is far too early for this to happen and as a consequence the sperm are severely reduced in their ability to move and have a major defect in the structure of their tail. We provide evidence that the reason this has happened is due to a dysregulation of calcium transport. This work is important for understanding defensin gene function in a living organism and may enable the design of novel contraceptives with additional antibiotic ability.

## Introduction

β-defensins are cationic peptides with a canonical six cysteines in their mature secreted peptide that were first isolated as antimicrobials and their presumed function is host defence. The β-defensin gene family consists of 40 family members at 5 gene loci in human and more than 50 genes over 4 loci in the mouse [1], [2]. The main cluster is on chromosome 8 in both human and mouse with 10 and 31 β-defensin genes respectively. In human, seven of the chromosome 8 genes lie at two distinct loci approximately 5 Mb apart as a highly copy number variable (CNV) cluster, which vary between 2 and 7 copies per genome [3]. Increased copy number above the mean number of 4 has been associated with increased risk of psoriasis [4]. It is evident that the evolutionary history of this gene family is complex with evidence for both rapid positive as well as negative selection [5]. The functional repertoire of β-defensin peptides has expanded recently to include involvement in pigmentation, immune cell attraction and immunomodulation [6]. However, the physiological function of mammalian β-defensins *in vivo* has not been determined.

β-defensins are highly expressed under normal conditions in different regions of the epididymal epithelia (see http://mrgd.org/index.cgi & [7]–[10]). They are secreted into the lumen and have been shown to be present on the plasma membrane of sperm [9], [11], [12]. It seems likely that they are involved in reproductive function and a few studies suggest that β-defensins influence sperm motility. The rat β-defensin Bin1b (SPAG11or EP2) has been shown to induce immature and immotile sperm to become progressively motile *in vitro* [9]. In addition, the β-defensin *DEFB126* on chromosome 20 has recently been linked to the ability of sperm to penetrate hyaluronic acid gel (a mimic of female cervical secretions). Men homozygous for a frameshift mutation in *DEFB126* are not infertile, but have reduced chance of successful fertilisation in the first year [13]. DEFB126 is quite different to other β-defensins, as it has an extensive C-terminal tail containing O-linked glycosylation sites that are not seen in other defensins. It is presumed this glycosylation is important for its function. Additionally in the rat, incomplete knockdown of Defb15 suggests that this peptide influences sperm motility, but not the capacitation process or acrosome reaction (AR) [12].

**Figure 1:**
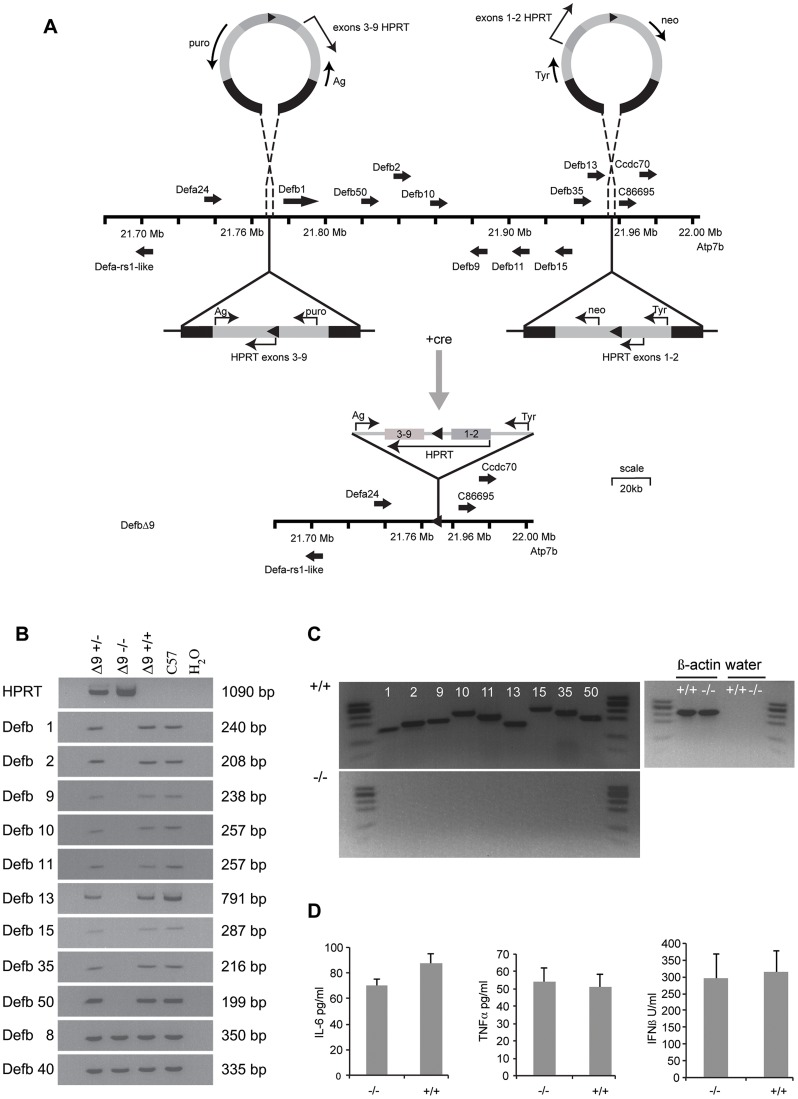
*DefbΔ9/DefbΔ9* mice with deletion of 9 β-defensin genes. Figure 1A: Generation and gene targeting of *DefbΔ9* mice. MICER vectors target two regions of the chromosome 8 β-defensin cluster in embryonic stem (ES) cells. Gene orientation indicated with solid arrows. First targeting vector carrying neomycin resistance (*neo*) and *Hypoxanthine-guanine phosphoribosyltransferase* (*HPRT*) exons 1 and 2 and *tyrosinase* (tyr), second targeting vector carrying puromycin resistance gene (*puro*), *Agouti* (*Ag*) and exons 3–9 of *HPRT*. DNA homologous to the genomic region indicated by black within the vectors. Following cre recombinase treatment of the double targeted cells recombination between the lox sites (solid triangles) results in a functional *HPRT* gene and E14 cells (normally *Hprt* mutant) that can now grow on hypoxanthine aminopterin thymidine (HAT) medium but are both puromycin and G418 sensitive. Further details in Figure S1. Vectors not drawn to scale. Figure 1B: PCR analysis of genomic DNA from *DefbΔ9* heterozygous (+/−), homozygous (−/−) and wild-type (+/+) mice for the nine β-defensin cluster deletion. Additional 2 positive control defensins not part of the deletion (*Defb8* and *40*) but in the centromeric chromosome 8 β-defensin cluster were included. DNA from C57Bl6 mice (C57) and water (H_2_O) are used as positive and negative controls respectively. DNA fragment sizes in base pairs (bp) are indicated. Figure 1C: RT-PCR analysis of 9 cluster defensin genes. RT-PCR analysis showing that *Defb1*, *2*, *9*, *10*, *11*, *13*, *15*, *35* and *50* mRNA are present in the epididymis of wild-type (+/+) mice (upper panel) but not present in *DefbΔ9* (−/−) deletion mice (lower panel). φx174 digested with *HaeIII* is used as a marker. Figure 1D: Levels of serum cytokines and type I interferon. Cytokine levels and type I interferon in *DefbΔ9* (−/−) and wild-type littermate sera as a marker for infection, do not deviate from the low levels expected in the absence of infection. Genotype groups are not significantly different (n = 4).

Single gene deletion of *Defb1* on chromosome 8 in mice has led to animals with a subtle gross phenotype, leading to the assumption that functional redundancy may reduce the severity of the expected phenotype [14], [15]. In order to address this issue and ascertain *in vivo* function, we aim to use gene targeting and *lox/cre* MICER (Mutagenic Insertion and Chromosome Engineering Resource) technology to selectively delete the β-defensin gene clusters in the mouse [16], [17]. In this study, we describe deletion of nine genes from the main thirty one β-defensin gene cluster on chromosome 8 (Figures 1A and S1). These are *Defb1*, *Defb50*, *Defb2*, *Defb10*, *Defb9*, *Defb11*, *Defb15*, *Defb35* and *Defb13*, which are the nine most telomeric genes of the cluster found adjacent to the intestinal α-defensin (cryptdins) genes. *Defb1*, *Defb15*, *Defb35* and *Defb13* are orthologous to the human genes *DEFB1*, *DEFB106*, *DEFB105*, and *DEFB107* respectively, but *Defb2, 10, 9, 11* (closely related paralogues) and *Defb50* are in murine restricted clades [5]. All nine deleted genes and their human orthologues are most strongly expressed in the male reproductive tract [18].

## Results and Discussion

### Generation of *DefbΔ9/DefbΔ9* mice

The gene targeting strategy used to delete the genomic DNA encompassing the nine β-defensin genes is shown schematically in Figure 1A and described in detail in Materials and Methods and Figure S1.The genotypes of the offspring derived from intercrosses of heterozygous mice carrying the 175 kb *DefbΔ9* nine gene deletion were obtained at the expected Mendelian frequencies of 1∶2∶1 for wild-type, heterozygous and homozygous mutant animals (data not shown). PCR analyses on genomic DNA or RT-PCR on cDNA synthesised from RNA from epididymides demonstrate the presence and expression of the nine deleted defensin genes in both heterozygous and wild-type mice, but their absence in homozygous mutant animals (Figure 1B and C). Importantly, genes that are expressed in the epididymis and are adjacent to, but outside the deletion (including *Bin1b* and *Defb33*), were not altered in their expression level (Figure S2).

The *DefbΔ9*/*DefbΔ9* mice homozygous for the deletion have no obvious gross phenotype compared to littermate controls after 8 generations of backcrossing to C57Bl/6. No change in size, weight or appearance is apparent, except mice carrying the mutant allele have paler tails by virtue of the *Agouti* transgene included in the MICER vector (data not shown). β-defensins are classically known as antimicrobial peptides, so we tested the mature peptides *in vitro* against a Gram-negative bacterium (*Pseudomonas aeruginosa, PAO1*). Table 1 illustrates the sequences of the mature synthetic peptides and Figure S3 reveals that all the peptides except from Defb15 are strong antimicrobials in either oxidised or reduced form. The active antimicrobials include Defb50, which does not have the canonical six cysteines and is missing the second cysteine of the motif (Table 1). Defb50 has poor antimicrobial activity in its oxidised form, but this improves under reduced conditions (Figure S3). These results support recent work by Schroeder et. al. (2011), which suggests that some β-defensins display improved activity following reduction [19]. Despite deleting the expression of these antimicrobials from the homozygous mice, there is no indication that the mutant mice have an increased inflammatory profile under normal animal housing conditions. There is no elevation in levels of TNF-α or IL-6 or type I interferon in sera from mutant versus wild-type mice (Figure 1D).

**Table 1:** Peptide sequence of β-defensins deleted in DefbΔ9 deletion.

Peptide	Signal sequence	Mature peptide
**Defb1**	**MKTHYFLLVMICFLFSQMEP**G	---VGILTSLGRRTDQYK**C**LQHGGF**C**LRSS**C**PSNTKLQGT**C**KPDKPN**CC**KS
**Defb50**	**MKTLCFLLLTSGLLYLMVK**GVGS	--------HPGTFHVRIK**C**MPKMTAVFGDN**C**SFYSSMGDL**C**NNTKSV**CC**MVPVRMDNI
**Defb2**	**MRTLCSLLLICCLLFSYTTP**	--AVGSLKSIGYEAELDH**C**HTNGGY**C**VRAI**C**PPSARRPGS**C**FPEKNP**CC**KYM
**Defb9**	**MRTLCSLLLICCLLFSYTTP**AANS	---------IIGVSEMER**C**HKKGGY**C**-YFY**C**FSSHKKIGS**C**FPEWPR**CC**KNIK
**Defb10**	**MRTLCSLLLICCLLFSYTTP**	--AVGDLKHLILKAQLTR**C**YKFGGF**C**HYNI**C**PGNSRFMSN**C**HPENLR**CC**KNIKQF
**Defb11**	**MRTLCSLLLICCLLFSYTTP**AVG	-----DLKHLILKAQLAR**C**YKFGGF**C**YNSM**C**PPHTKFIGN**C**HPDHLH**CC**INMKELEGST
**Defb35**	**MPQTFFVFCFLFFVFL**QLFPGTG	-----------EIAV**C**ET**C**RLGRGK**C**RRA-**C**IESEKIVGW**C**-KLNFF**CC**RERI
**Defb15**	**MKTFLFLFAVLFFLDP**AKNAF	--------------FDEK**C**SRVNGR**C**TAS-**C**LKNEELVAL**C**-QKNLK**CC**VTVQP**C**GKSKSNQSDEGSGHMGTWG
**Defb13**	**MRIFSLIVAGLVLLIQLYP**AWG	-----------TLYRRFL**C**KKMNGQ**C**EAE-**C**FTFEQKIGT**C**-QANFL**CC**RKRKEH

Single letter amino acid sequence of the predicted peptide encoded by the β-defensin genes in the *DefbΔ9* deletion. The signal sequence is separated from the mature peptide sequence as determined by ExPASy proteomics tool http://web.expasy.org/peptide_cutter/. These are two exon encoded genes, and the first exon encoded amino acids are in bold, second exon encoded amino acids are non-bold. Mature peptide sequences are aligned using the classical 6 cysteine motif (present in all but Defb50) and spaces are introduced (marked by -) to enable this. Cysteines in the mature peptide are emboldened.

### *DefbΔ9/DefbΔ9* homozygotes are infertile and their sperm have reduced motility

**Figure 2:**
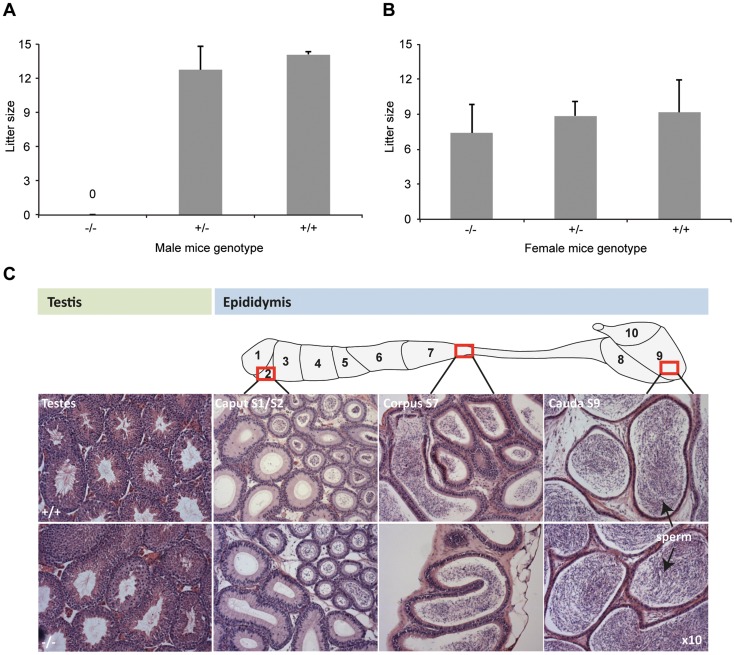
*DefbΔ9/DefbΔ9* male mice are infertile. Figure 2A: Litter sizes of *DefbΔ9/DefbΔ9* (−/−) male mice mated to wild-type CD1 females over 3 months. The homozygous mutant males produced no litters. Figure 2B: Litter sizes of DefbΔ9 female mice mated to wild-type CD1 males over 3 months. *DefbΔ9* (−/−) female mice reproduced normally with no significant difference in litter sizes. The variation in litter size for the wild-type and heterozygous mice between panel A and B is due to the genetic background difference between the females used (CD1 in panel A and C57Bl/6 in panel B). Figure 2C: Histology of testis (left) and epididymis (right) of wild-type (+/+) (top panel) and *DefbΔ9/DefbΔ9* (−/−) (bottom panel) mice. Tissue from approximately 5 months old mice were fixed in bouin's fixative, paraffin wax blocked, cut at 7 µm thick sections and stained with H&E stain. No obvious histological difference is present between the wild-type and mutant tissue at the light microscopy level. Sperm are easily visible (arrowed) in both wild-type and mutant cauda. Original magnifications ×10.

The breeding of homozygous *DefbΔ9* males to wild-type CD1 females reveals an inability to produce offspring (Figure 2A), but the homozygote mutant females have comparable fecundity to wild-type and heterozygote littermates when mated to CD1 males (Figure 2B). The male phenotype is not sperm-cell autonomous, as heterozygous male mice when crossed to wild-type females produce similar numbers of wild-type and heterozygous offspring (56 heterozygotes and 61 wild-type). This demonstrates that haploid sperm cells with the mutant allele are not disadvantaged compared to sperm carrying the wild-type allele when produced in *DefbΔ9* heterozygous mice.

Despite the inability of mutant male mice to reproduce, the tissue histology of testis and epididymis shows no obvious structural abnormalities or differences from wild-type littermates at 5 weeks, 10 weeks or 20 weeks. Testes are not significantly altered in weight (data not shown), and spermatogenesis appears normal with sperm being produced and subsequently stored in the epididymides (Figure 2C).

**Figure 3:**
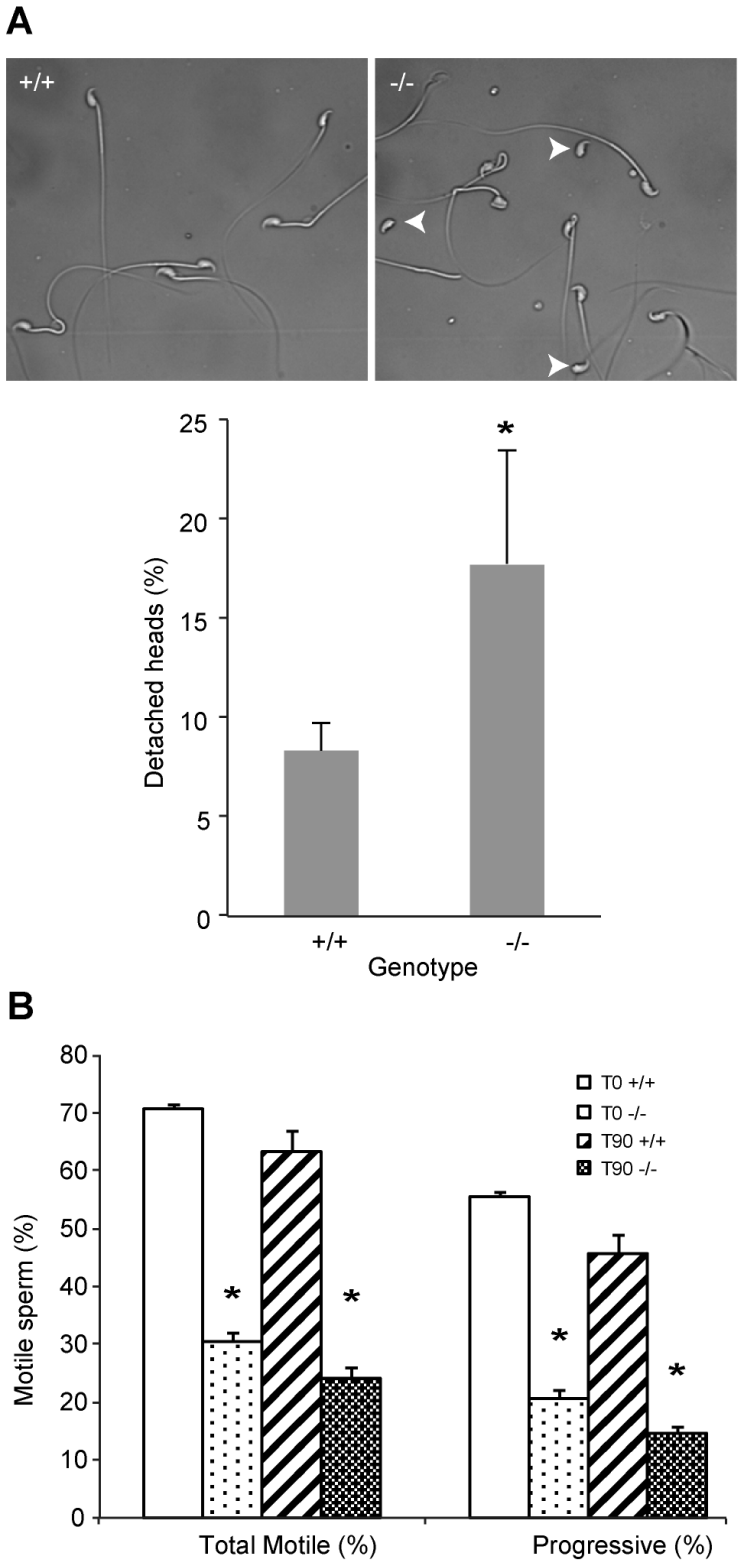
*DefbΔ9/DefbΔ9* male mice have more fragile sperm with reduced motility. Figure 3A: Cauda epididymal sperm from *DefbΔ9/DefbΔ9* (−/−) show an increased number of detached heads compared to wild-types (+/+). Photomicrographs represented are in phase-contrast microscopy at original magnification of ×40. Fragility of the mutant vs wild-type derived sperm isolated from cauda was determined by dropping the suspensions onto a glass slide and analysing the number of detached heads (arrowheads). A total of 200 sperm were analysed for each slide, which represented one animal. An average of 3 pairs was analysed (mean ± SD; n = 3). p = 0.048. Figure 3B: Spermatozoa from *DefbΔ9/DefbΔ9* mice have reduced motility. Percentage of total (left) and progressive (right) spermatozoa motility in *DefbΔ9* +/+ and *DefbΔ9* −/− mice using CASA before (time 0 mins, T0) and after (time 90 mins, T90) sperm capacitation (mean ±SD; n = 4). *, p<0.001.

Epididymal sperm cells from the cauda of homozygous mutant animals were present in similar numbers to those from wild-type animals. However, the mutant derived sperm are more fragile compared to sperm from wild-types, resulting in significantly higher numbers of headless tails when exacerbated by dropping the sperm suspension onto a glass slide (Figure 3A).

In mammals, ejaculated sperm need to complete capacitation before being competent to fertilize a mature oocyte. This process occurs in the female reproductive tract. It involves several changes in membrane properties and an increase in intracellular calcium that drives motility and induction of the AR [20]. Only capacitated sperm can bind glycoproteins of the zona pellucida (ZP), undergo AR and fertilize a mature oocyte.

Sperm were freshly isolated into modified Tyrode's complete medium to induce sperm capacitation [21] and subjected to analysis using computer assisted sperm analyses (CASA) at various time points. Spinning or vigorous pipetting was avoided to minimise any *ex vivo* effects on sperm viability and/or motility. The sperm from the mutants have a very obvious and significant lower percentage of progressive motility both before capacitation at time 0 minutes (T0) and after capacitation induction at time 90 minutes (T90) (Figure 3B).

### Sperm from *DefbΔ9/DefbΔ9* mice are prematurely acrosome reacted

**Figure 4:**
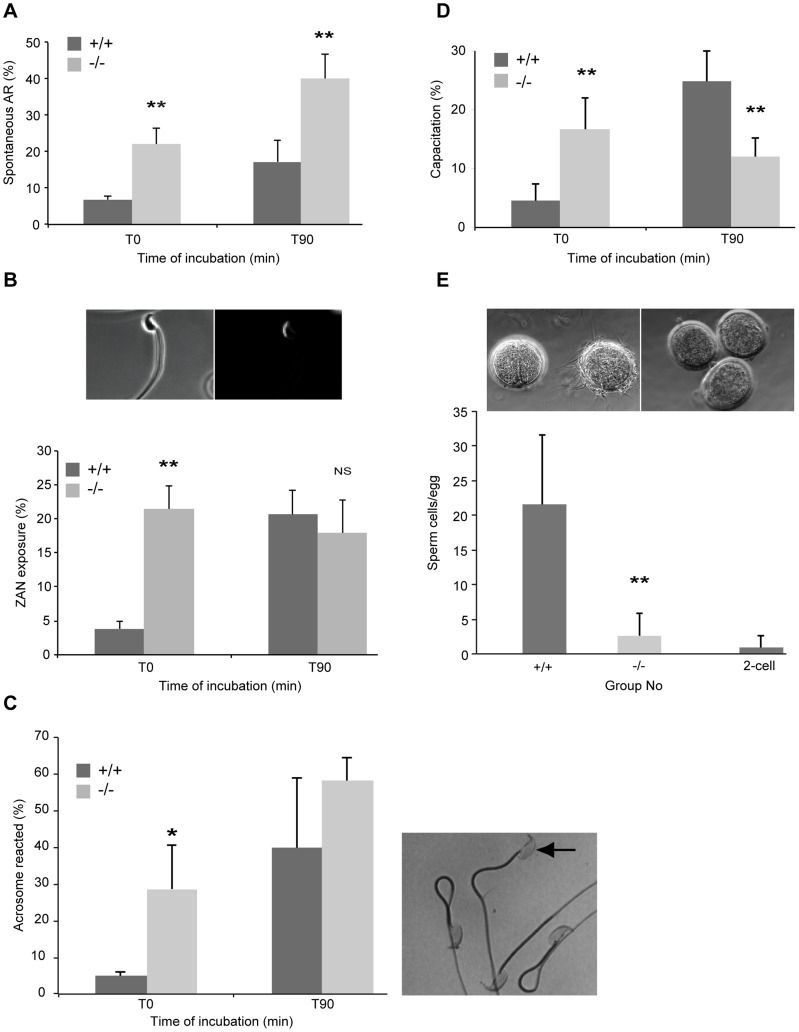
Precocious capacitation and increased spontaneous acrosome reaction in sperm from *DefbΔ9/DefbΔ9* mice. Figure 4A: FITC-conjugated Pisum sativum (PSA) lectin labelling of acrosome reacted sperm. Percentage of spontaneous AR in wild-type (+/+) and *DefbΔ9/DefbΔ9* (−/−) sperm determined by PSA-FITC lectin labelling before (T0) and after 90 minutes incubation (T90) in complete capacitation medium (mean ±SD; n = 4). *, p<0.05. Figure 4B: Zonadhesin antibody binding and quantification. Images show representative ZAN antibody binding to a sperm from *DefbΔ9* (−/−) male after capacitation, visualised with a goat anti-rabbit IgG conjugated to Alexa Fluor 594 around the sperm head. Left panel (bright field) and right panel (fluorescent image). Lower panel shows percentages of ZAN exposure evaluated on live spermatozoa from +/+ and −/− before (T0) and after (T90) incubation in capacitation medium. Figure 4C: Acrosome integrity following Coomassie blue G250 staining of fixed sperm. Right panel shows representative image of sperm that is acrosome reacted (arrow) lacking staining and other non-AR sperm with intense staining. Left panel graph shows average percentage of acrosome reacted sperm from three independent experiments where over 150 sperm were counted per sample before and after incubation in complete capacitation medium (mean ± SD; n = 3). p<0.03. Figure 4D: Percentage of capacitation evaluated by the ability of sperm to undergo the AR. AR induced by 10 µM calcium ionophore A23187 in spermatozoa from mutant (−/−) and wild-type (+/+) animals and level of PSA-FITC used to determine AR directly after the treatment (T0) and 90 minutes after (T90) (mean ±SD; n = 4). *, p<0.01. Figure 4E: Sperm-egg binding assay. Light microscopy images show cumulus-free eggs from superovulated CD1 females with sperm from wild-type animals (+/+) (upper left panel) and no sperm from *DefbΔ9−/−* males bound to the eggs (upper right panel). Sperm were also incubated with 2-cell embryos as a control for non-specific binding (left egg in upper left panel). A range of 47–86 eggs per genotype were used for each set of experiments (n = 3). Original magnification ×20. Graph shows comparison of the average number of sperm from wild-type (+/+) and *DefbΔ9* (−/−) males bound eggs following 45 minute incubation (mean ± SD; n = 3). **, p<0.001.

We determined the capacitation and AR state of the mutant and wild-type sperm to ascertain whether the maturity of the sperm was altered. *Pisum sativum* (PSA) lectin binds to the outer acrosomal membranes of the sperm head and loss of binding indicates that the sperm cells have undergone the AR [22]. We find reduction in the ability of PSA-FITC to bind the *DefbΔ9/DefbΔ9* derived sperm directly after dispersal from the cauda (without induction of capacitation), indicating significant increase in spontaneous AR (20% for *DefbΔ9/DefbΔ9* derived sperm versus 8% for wild-type) (Figure 4A). At time 90 minutes, after sperm capacitation there are twice as many acrosome-reacted mutant sperm compared to wild-type derived sperm cells (Fig. 4A).

We confirmed the above result using a recently described measure of the ability of sperm to successfully fertilize. Tardif et. al. demonstrated that zonadhesin (ZAN) epitopes are only revealed by AR when the sperm are competent to undertake fertilization [23]. We observe that ZAN was already exposed (and able to bind to an antibody against ZAN) on 20% of the sperm from the *DefbΔ9* mutant mice immediately after isolation from the cauda (T0), and this percentage does not increase over time in capacitation medium (Figure 4B). In contrast, the sperm from wild-type animals show a continuum of ZAN exposure, from 3% at T0 to a maximum level of 22% after 90 minutes incubation in capacitating medium (Figure 4B).

Coomassie blue G250 stains the acrosome of the sperm. This technique allows direct visualisation of the acrosome or its absence following AR under light microscopy. Analysis and quantification is determine by scoring at least 150 sperm with the presence or absence of an intense blue stain on the anterior sperm head [24]. The results show that the mutant sperm have significantly increased and premature spontaneous AR with 28% of mutant sperm showing AR compared to 5% of wild-type sperm at T0 (Fig. 4C). This mirrors and further supports the PSA lectin and ZAN exposure assessments of AR.

No direct procedure is available to determine capacitation status, but AR induction informs on the rate of sperm capacitation. Therefore, rate of sperm capacitation can be evaluated indirectly by measuring the number of cells without an acrosome following induction of the AR, as only capacitated cells can undergo this process. We find that the rate of capacitation is very different between sperm from wild-types and homozygotes. The mutants display the optimal percentage of capacitation at T0 as estimated by measuring AR by PSA-FITC binding, following calcium ionophore induced capacitation and AR. This level does not increase after 90 mins whereas the status of sperm capacitation for wild-type derived sperm increases from 6% to 23% over this same period of time (Figure 4D).

One might expect that sperm that are prematurely capacitated may bind to the zona pellucida (ZP) of oocytes more effectively than wild-type sperm. Paradoxically the sperm from the mutants are extremely poor at binding firmly to the ZP of eggs, whereas wild-type sperm bind effectively (Figure 4E). Recent studies in the mouse have shown that sperm that have undergone the AR can penetrate an egg, although this was not an efficient process [25], [26] and mouse sperm from several KOs cannot strongly bind to the ZP and yet are still able to fertilize [27], [28].

### Sperm from *DefbΔ9/DefbΔ9* mice have disrupted microtubule structure

**Figure 5:**
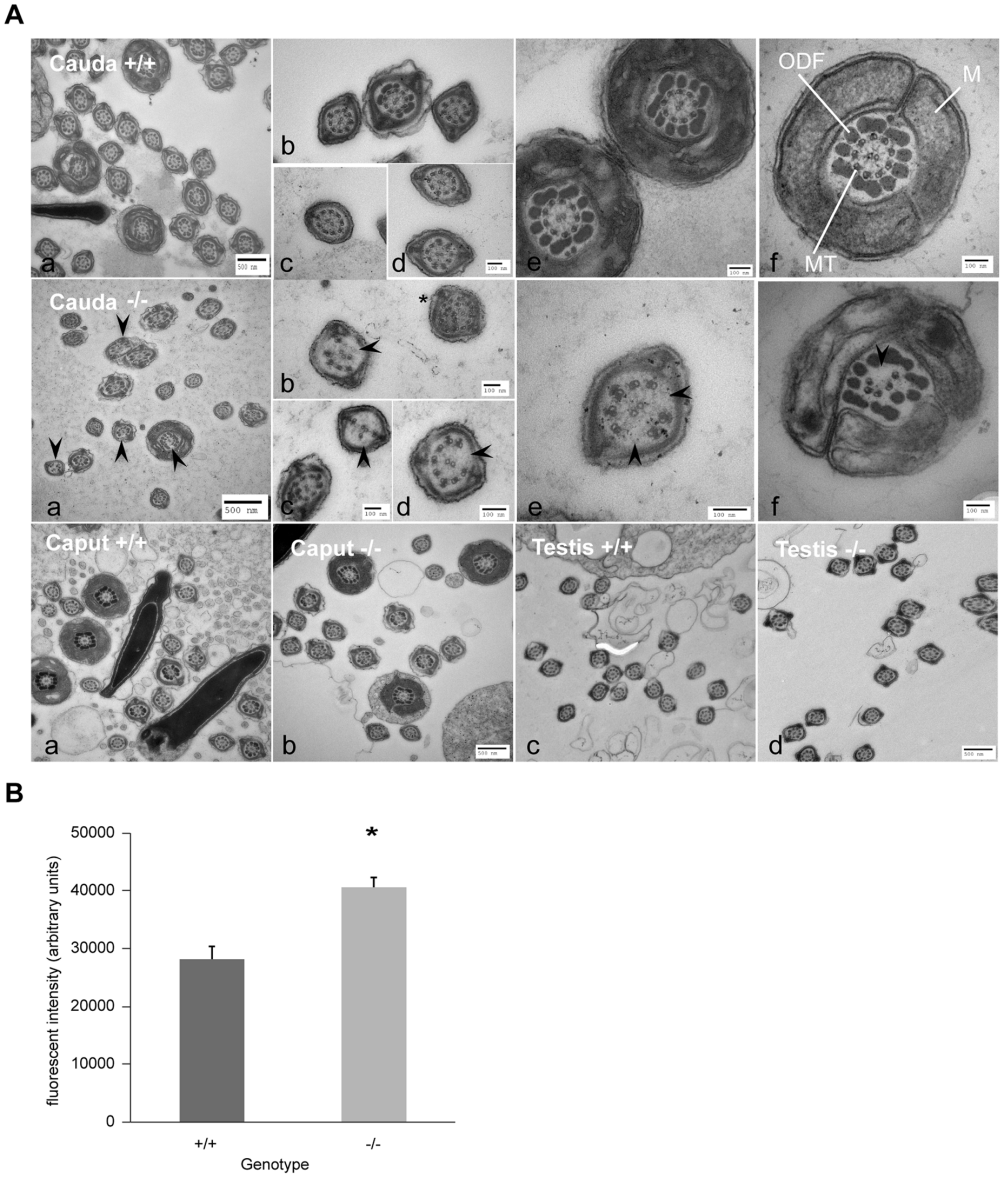
Ultrastructure of spermatozoa from cauda, caput and testis from wild-type littermates (+/+) and *DefbΔ9/DefbΔ9* (−/−) male mice reveals a defect in microtubule structure. Figure 5A: Transmission Electron Microscopy (TEM) of sperm from cauda, caput and testis from *DefbΔ9/DefbΔ9* (−/−) and wild-type littermates (+/+) mice. Top and middle horizontal panels show overviews of cross section of sperm from cauda of +/+ and −/− mice respectively at various levels of the tail (a). Higher magnification of cross section of principal piece (b–d) and mid-piece (e–f) of sperm tails. Upper panel (f) shows normal axoneme (9+2 microtubules, MT), mitochondrial sheath (M), outer dense fibres (ODF) in wild-type mouse sperm. Middle panel (f) shows clear disruption and disintegration of the MT (arrowhead) in sperm from *DefbΔ9* (−/−). Middle panel (b) shows example of additional microtubules (*) other than the classical 9+2 arrangement. Bottom panel shows TEM of caput epididymal (a, b) and testis (c, d) sperm from wild type (+/+) and *DefbΔ9*(−/−) mice. No obvious microtubule disruptions were observed in sperm within the caput or testis of the mutant mice. Bars: 500 nm and 100 nm as labelled (a–f). Figure 5B: Fluorescent intensity of total intracellular calcium of wild-type (+/+) littermates and *DefbΔ9/DefbΔ9* (−/−) sperm using Fluo3 AM ester assay. The calcium levels were measured using Fluo-3 AM ester calcium fluorescent indicator at 5 µM concentration incubated with spermatozoa at 20 million/ml for 30 mins at 37°C, samples were washed and loaded onto 96-well plate in duplicates at 100 µl/well, and the plates were read by BMG Labtech FluoSTAR Omega fluorescent reader (mean ± SD; n = 3).*, p<0.002. Values given are after subtracting the background levels of the DMSO controls.

Ultrastructural analyses using transmission electron microscopy (TEM) reveals an abnormally high number of cells with disruption of the classic 9+2 microtubule arrangement in the tail axoneme of sperm from mutants compared to the sperm from wild-type littermates (41% for −/− vs 4% for +/+) (Figure 5A and data not shown). To reduce potential artefacts introduced by processing of purified sperm cells, we analysed tissue samples from testes, caput and cauda, and examined the structure of the sperm tails still within these tissues. These analyses reveal that sperm present in the cauda (but not in caput or testes) of *DefbΔ9/DefbΔ9* mutants show an increase in disruption of the microtubule structure, where the 9+2 arrangement has disintegrated (Figure 5A). This phenotype is reminiscent of sperm cells that have undergone hyperactivation following capacitation, where increased tail movement can result in disintegration of the axonemal filaments in sperm that are demembranated and stimulated with calcium [29]. A very similar microtubule disruption phenotype is also seen in sperm of mice with deletion of the group III secreted *phospholipase A_2_* (*sPLA_2_-III*), and like the *DefbΔ9* mutants this is only present in sperm cells isolated from the cauda [30]. Interestingly, secreted phosphlipase A2 enzymes have a cysteine rich structure and like defensins have antimicrobial activity [31].

### Increase in intracellular calcium content in sperm from *DefbΔ9/DefbΔ9* mice

We sought to understand why sperm from the *DefbΔ9/DefbΔ9* have this precocious capacitation and increased spontaneous AR. Sperm require an increase in intracellular calcium, which causes hyperactivation and allows progression to the AR. Mice deleted for any of the components of the calcium CatSper (cation channels of sperm) channel are infertile due to their lack of ability to transport calcium into the cell, resulting in an inability to undergo capacitation, hyperactivation and prepare sperm for the AR [32]. Conversely, sperm exposed to a ten-fold increase in cauda epididymal calcium concentration in mice mutant in *TRPV6* (transient receptor potential vanilloid 6) that is a calcium ion selective channel, display increased intracellular calcium and have a markedly reduced motility and fertilization capacity [33]. In addition, sperm induced with 10 µM of the calcium ionophore A23187 will undergo the AR, but will become immotile [34].

We treated sperm from wild-type littermates (+/+) with A23187 to release calcium and induce AR, and examined their ultrastructure by TEM at the T90 minutes time point (Figure S4). The A23187 treatment resulted in 53% of these wild-type sperm being acrosome reacted as judged by Coomassie G250 stain (157 sperm undergone AR out of 293), compared to 14% (38 sperm undergone AR out of 271) of the untreated wild-type controls. The TEM revealed that a phenotype of microtubule disruption was evident in 52% (101/195 tails in focus) of the A23187 treated sperm compared to 3% (3/105 tails in focus) in the controls (Figure S4). This strongly suggests that excessive intracellular calcium may induce microtubule defects similar to those we observe in the *DefbΔ9* mutant sperm.

We determined the intracellular calcium concentration of freshly isolated sperm from both *DefbΔ9/DefbΔ9* and wild-types to see if this could explain the increased spontaneous AR and microtubule defect present in the mutants. The sperm from mutant animals show significantly increased calcium concentration compared to wild-type littermates (Figure 5B). The altered calcium concentration does not reflect increased numbers of non-viable sperm, as sperm killed using heat, demonstrate only a low background level of intracellular calcium comparable to the negative vehicle-treated control (data not shown). Thus, in the absence of the deleted β-defensins, there is significant increase in intracellular calcium (Figure 5B) and the likely consequence of this is an increase in spontaneous AR and microtubule disruption. Defensins have diverse receptor-binding activity [6], and pertinently the defensin-like molecule MsDef1 from Alfalfa seed has been shown to have the ability to block mammalian L-type calcium channel activity [35]. It is therefore possible that the rise in calcium that we observe is due to the lack of β-defensin(s) from the membrane, allowing transport of the ion through the CatSper (or other) calcium channel. In wild-type cells this does not happen until the membrane remodelling occurs during capacitation. Recent work on the secreted seminal vesicle protein SPINK3 supports this idea [36]. SPINK3 has calcium transport inhibitory activity and when added to capacitated mouse sperm, the number of acrosome reacted sperm is significantly lower compared to sperm not exposed to this peptide. The implication of this is that sperm in the male reproductive tract is inhibited from undergoing the AR until near the egg [36]. β-defensins may act as an additional protection against inappropriate activation of sperm in the epididymis, a site where sperm are mature but not placed for fertilization.

Taken together, the results from the deletion mice, demonstrate for the first time that β-defensins are important for suppression/regulation of spontaneous AR and are essential for fertility. The sequence of the mouse genome (GRC m39) reveals that the region we have deleted specifically contains only these nine β-defensin genes and no other annotated feature expressed in the male reproductive tract are found. However, we do not know which of the deleted gene(s) in the cluster are responsible for the unique fertility phenotype. It is suggested that epididymal maturation of sperm cells occurs most likely in the caput or corpus region of the epididymis rather than the cauda [9]. Of the 9 genes, only *Defb15*, *Defb35* and *Defb13* are strongly expressed in these regions with the others being expressed predominantly in the cauda [10]. We have evidence from mass spectrometry analyses that Defb2, Defb11 and Defb15 are present on isolated wild-type cauda sperm (data not shown), but have not detected the other peptides. Rat Defb15 binds the acrosomal region of caput sperm, and incomplete knockdown results in rats with reduced sperm motility, but no defect in capacitation or AR [12]. Interestingly, Defb15 has an extended carboxyl tail containing an extra cysteine and six potential serine or threonine residues that would support O-linked glycosylation, which is considered a key feature of the function of DEFB126 in human sperm [13]. However, the motility defect described in the sperm isolated from *DEFB126* homozygous men is quite different to the phenotype we describe here. Unlike Tollner's study, where the sperm from homozygous men only display abnormal motility when tested in the cervical mucus mimic using viscous hyaluronic acid, our *DefbΔ9*/*DefbΔ9* derived sperm have an obvious motility defect even in isolation medium.

It may be that due to the deletion of several genes we are observing a compound or additive phenotype, indeed deletion of a single gene might not demonstrate a strong enough phenotype to be easily recognised. The three human orthologues to *Defb15*, *Defb35* and *Defb13* (*DEFB106*, *DEFB105* and *DEFB107* respectively) are on the chromosome 8 8p23.1 highly CNV block [3]. In primates, protection against loss of fertility from mutation might be a selective advantage that allows the increase in copy number of these genes to be maintained [37]. Pertinently, human sperm samples with a high spontaneous AR may have significantly lower fertilisation rate by in vitro fertilization (IVF) compared to samples used with a normal and low AR [38].

For the first time, we show here that β-defensin genes have a profound effect on sperm function *in vivo*, and this is manifested in the *DefbΔ9/DefbΔ9* mice by increased intracellular calcium, precocious capacitation and increased spontaneous AR, which results in microtubule destabilisation, lack of motility and profound infertility. This provides evidence for the β-defensin(s) in this cluster being essential for control of intracellular calcium and regulation of AR. This improved understanding of the function of these antimicrobial peptides leads the way not only towards increased understanding of male infertility, but also the development of novel and highly effective contraceptives with additional antimicrobial action for local use in the female reproductive tract.

## Materials and Methods

### Animal studies

Animal studies were performed under UK Home Office license and permission and local ethical approval. The *DefbΔ9* mice used in the studies were derived from C57BL/6N and 129 strain background, subsequently backcrossed to C57BL/6N for at least 4 generations.

### Generation of DefbΔ9 mice

We chose to use the *lox/cre* double targeting strategy described originally by Adams *et. al.* [16], and used successfully in several reported studies to introduce precise deletions of the genome [17]. The defensin cluster on chromosome 8:A1–A2 in the mouse consists of 31 β-defensin or α-defensin-like genes from 8∶18,974,940 to 8∶20,922,071. Within the cluster is an expansion of genes from 8∶21,025,545–8∶21,735,471 that are derived from the β-defensins and are termed α-defensins (cryptidins) due to their different cysteine spacing and connectivity [39]. There are nine β-defensin genes telomeric to the cryptidins and these are the genes we deleted. A MICER clone carrying exons 1 and 2 of the *HPRT* gene, a *neo* selection cassette and the *Tyrosinase* gene and 7 Kb of homology to the genomic region downstream of *Defb13* was constructed in house and was linearized with *SalI* and electroporated into *129/Ola* E14(IV) cells (kind gift of Austin Smith). Targeted clones were isolated with a long range PCR from vector DNA to genomic DNA outwith the vector and hybridised to an internal oligonucleotide to validate the PCR fragment (Figure S1A). Clones were isolated at a low frequency of 1 in 203. This clone was then subjected to a second round of targeting to the region upstream of *Defb1* using the MICER clone MHPP423o12, which has 9 Kb of homology to the mouse genome and carries the *HPRT* exons 3–8 and *puromycin* selection gene. Correctly targeted clones were isolated at a high frequency of 1 in 4 (Figure S1B) and correctly targeted clones were isolated for cre recombinase treatment and selection in HAT. Only clones that undergo the *lox* site-mediated recombination in the presence of cre create a functional *HPRT* gene that will allow the growth of the *HPRT* mutant E14 cells in HAT selection. Some clones produced HAT resistant clones at a frequency that was at least 10 fold lower than other clones. We presumed this was due to intra versus inter-chromosomal recombination as described previously indicating that the targeting events were on the same chromosome [16]. We isolated HAT resistant clones from targeted cell lines that were most efficient at producing colonies after cre exposure and selection. As expected, these HAT resistant clones were now puromycin and G418 sensitive, as the plasmid sequences containing these selection cassettes were lost during the recombination (Figure 1A) and PCR of the *HPRT* gene was successful and showed sequence consistent with the expected *lox*-mediated recombination event (Figure S1C).

### Polymerase Chain Reaction (PCR) analysis

PCR analysis of genomic DNA isolated from tail tips from *DefbΔ9* mice for the 9 defensin cluster deletion were carried out using the sequence specific mouse primers as in Table S1. The primers were used at a final concentration of 0.2 µM each in the PCR reaction, which were carried out under standard conditions using Platinum Taq polymerase (Invitrogen). PCR products were visualised on ethidium bromide stained 2% agarose gels.

### RNA extraction

Total ribonucleic acid (RNA) was extracted from cells using the method of Chomczynski and Sacchi [40].The epididymides were removed from mutant and C57Bl/6 mice, homogenised in 1 ml of RNAzol (Biogenesis, Dorset, U.K.) in a 2 ml RNAse free tube (Sarstedt, Leicester, U.K.), into which 100 µl of chloroform was added, vortexed and left on ice for 5 mins. Following centrifugation at 10,000 rpm for 15 mins, the upper aqueous layer was removed into a fresh tube and an equal volume of ice-cold isopropanol added. The solution was mixed and left at −20°C for 30 mins. The RNA was then precipitated by centrifugation at 10,000 g for 20 mins. The resulting RNA pellet was washed twice with 75% ice cold ethanol (2×5 mins 10,000 rpm spins) and resuspended in 20–100 µl of RNAse free water. The concentration and purity of the RNA was determined by spectrophotometry (GeneQuant II, Pharmacia Biotechnology, St Albans, U.K.).

### Preparation of cDNA

Complementary DNA (cDNA) was made by the process of reverse transcription using a cDNA synthesis kit (Roche Applied Science). Briefly, 1 µg of RNA in a volume of 8.2 µl was reverse transcribed by mixing with the following components, 2 µl oligo dT primer (0.8 µg/µl), 2 µl reaction buffer (×10), 2 µl dNTP mix (40 mM), 4 µl 25 mM MgCl_2_, 1 µl RNase inhibitor (50 U/µl) and 0.8 µl reverse transcriptase (200 u/µl). The reaction was carried out at 25°C for 10 mins and then at 42°C for 60 mins. The tube was then placed at 95°C for 5 mins, after which time the cDNA was used for PCR.

### Polymerase Chain Reaction (PCR) and quantitative (q) RT-PCR

cDNAs were amplified using sequence specific mouse primers. Sequences of the primer pairs are shown below. The primers were used at a final concentration of 0.2 µM each in the PCR reaction, which were carried out under standard conditions using Platinum Taq (Invitrogen). The thermal cycling protocol for all primers comprised an initial denaturation step at 94°C for 2 minutes followed by 35 cycles of 94°C for 1 minute, 55°C (Vary according to primer set) for 1 and 72°C for 1.5 minute. The final cycle consisted of a re-annealing at 72°C for 10 minutes. PCR products were visualised on ethidium bromide stained 2% agarose gels.

Quantitative PCR was carried out using the Lightcycler 480 Real-time PCR System (Roche). Primers were designed using the Roche Universal ProbeLibrary Assay Design Center (www.roche-applied-science.com/sis/rtpcr/upl/index.jsp?id=UP030000). The reference gene used was the Universal ProbeLibrary Mouse GAPDH Gene Assay to allow for quantification of gene expression levels using dual-color real-time PCR. cDNAs were amplified using sequence specific mouse primers. The primers were used at a final concentration of 0.2 µM each in the PCR reaction, which were carried out under standard conditions using LightCycler 480 Probes Master (Roche Applied Science). The relative quantification of each target gene was determined using the expression levels of the reference gene. All samples were analysed in triplicate.

The primers and annealing temperature for PCR amplification of cDNAs and primer sets used for quantitative RT-PCR are given in Supplementary material S4.

### Sperm cell isolation

Caudal epididymides sperm were dispersed in modified Tyrode's medium [21] after mincing the cauda and incubating at 37°C (5% CO_2_) for approximately 15 minutes. Following cell dispersion and removal of tissue, sperm concentration was assessed by using a cell counter chamber.

### Sperm fragility assay

Fragility of the caudal epididymis sperm of mutant and wild-type mice was determined by dropping approximately 30 µl sperm suspension onto a microscope glass slide from a determined height using a ruler. A coverslip was placed on top and subsequently sealed with nail varnish. The number of intact sperm and detached heads were quantified. A total of 200 sperm were analysed for each slide, which represented one animal. An average of 3 pairs was analysed.

### Calcium assay

The calcium levels were measured using Fluo-3 AM ester (Molecular Probes F14218; Invitrogen) calcium fluorescent indicator (methodology adapted from [36]). One of the advantages of using Fluo-3 is that it exhibit large fluorescent intensity increases on binding calcium (typically >100-old). Unlike the ultra-violet light-excited indicators fura-2 and indol-1, there is no accompanying spectral shift. Caudal epididymis sperm were isolated as described above in calcium-free modified Tyrode's medium from sexually mature males ranging from 11–19 weeks old (mean age: 14.9 weeks). The respective wild-type and knockout male mice chosen for each set of experiment are all matched for age, diet, living conditions and are housed separately from female mice. After isolation, sperm aliquots at 20 million/ml concentration were transferred to pre-warmed 1.5 ml eppendorf tubes and incubated in the presence of 5 uM Fluo-3 AM (1∶200 dilution from 1 mM stock) or DMSO as control at 37°C for 30 mins.

Samples were washed by centrifugation (3×0.7 g for 5 mins) and loaded onto Black Greiner 96-well bottom plate (Sigma-Aldrich) in duplicates at 100 µl/well. Heat-killed sperm with Fluo3 AM ester and sperm with DMSO were used as controls. The plates were read by BMG Labtech FluoSTAR Omega fluorescent reader where the fluorescent intensity was measured using appropriate wavelength settings (excitation at 485 nm, emission at 520 nm). Fluorescent intensity of total intracellular calcium of *DefbΔ9* +/+ and *DefbΔ9* −/− sperm after subtracting the background levels of the DMSO controls were shown (mean ± SD; n = 3 pairs).

### Fertility breeding

Male *DefbΔ9* mice were set to breed with CD1 females mice, while female *DefbΔ9* mice were set up to breed with CD1males over a period of approximately 3 months. For each genotype 3–6 individual breeding pairs were set up, and the average pups per litter was calculated for both male and female mice.

### Sperm analysis with CASA

Caudae epididymides from *DefbΔ9* +/+ and −/− mice were isolated and placed in pre-warmed modified Tyrode's medium supplemented with 4 mg/ml BSA at 37°C (5% CO_2_) as described previously [21]. Aliquots of sperm were taken at 2 time points (T0 and T90 mins), diluted fourfold with the same media and placed in pre-warmed 80 µm glass chamber for computer-assisted sperm analysis (CEROS; Hamilton Thorne Biosciences Beverly, MA). For each animal, 3–4 microscope fields from each of the 2 chambers were video-recorded, capturing 200–400 sperm. Images were captured at 60 fps Hz for 30 frames and sperm parameters such as percentage of total motile, progressive motile, average path velocity (VAP), straight line velocity (VSL) and curvilinear velocity (VCL) were analysed.

### Transmission electron microscopy

Epididymides and decapsulated testis were fixed with 2% paraformaldehyde (PFA), 2.5% glutaraldehyde in 0.1M sodium cacodylate buffer + 0.04% CaCl_2_ for 30 mins at room temperature. The tissues were cut roughly into 1 mm cubes and further fixed overnight at 4°C. Fixed cells were rinsed in 0.1M sodium cacodylate buffer + 0.4% CaCl_2_, post-fixed in 1% osmium tetroxide (OsO4, Agar Scientific) for one hour, and dehydrated in sequential steps of acetone (25%, 50%, 75% and 100% twice) prior to impregnation in increasing concentrations (25%, 50%, 75%) of resin (TAAB Lab Equipment) in acetone followed by 100% resin for 3 times, placed in moulds and polymerised at 60°C for 24 hrs. Ultrathin sections of 70 nm were subsequently cut using a diamond knife on a Leica EM UC7 ultramicrotome. Sections were stretched with chloroform to eliminate compression and mounted on Pioloform filmed copper grids prior to staining with 1% aqueous uranyl acetate and lead citrate (Leica). They were viewed on a Philips CM100 Compustage (FEI) Transmission Electron Microscope with images collected using an AMT CCD camera (Deben).

### Sperm-egg adhesion assay

Sperm-ZP binding was assessed by a gamete co-incubation assay. Mouse oocytes and 2-cell embryos were collected from superovulated CD1 female mice into M2 media. The cumulus cells were removed from the oocytes by incubating the cumulus masses in M2 containing 1% hyaluronidase. The cumulus free oocytes were washed through several drops of M-199M (M199 media supplemented with 4% BSA and 30 µg/ml of sodium pyruvate) to remove any loose cumulus cells and any hyaluronidase media. They were then grouped with the 2-cell embryos in 50 µl microdrops of M-199M media, each group contains twelve oocytes and three 2-cell embryos, and put into the 37°C, 5% CO_2_ incubator until required.

The sperm from the caudal epididymides of wild-type (+/+) and *DefbΔ9*(−/−) mice were collected into 5 mls of M-199M and capacitated for 90 minutes at 37°C in 5% CO_2_. Approximately 5000 capacitated sperm (5 µl of 10^6^/ml sperm suspension) were added to each 50 µl microdrop containing the oocytes and 2-cell embryos as control and incubated for 45 minutes at 37°C in 5% CO_2_.

After this time the oocytes and 2-cell embryos were removed from the sperm drops into fresh M-199M media microdrops using a 120 µM diameter Pasteur pipette (Bio Medical Instruments) and were washed through several microdrops of media to remove the non-specific bound sperm. The oocytes and 2-cell embryos were transferred to microdrops of fixative made from 1∶1 mix of M199 media with 2% formaldehyde in PBS/PVP solution. This fixes the oocytes and 2-cell embryos with any sperm bound to them and allows the sperm bound to be counted and analysed.

### PSA lectin binding for capacitation and AR determination

Sperm proteins were detected in methanol-fixed and permeabilized mouse. Sperm capacitation of caudae epididymides was determined by the ability of sperm to undergo the acrosome reaction (AR) in the presence of calcium ionophore A23187 (Molecular Probes) as previously described Calcium ionophore A23187 or DMSO alone as vehicle was added to the sperm samples (10–20×10^6^ sperm/ml) at 10 µM concentration and incubated for 15 mins at 37°C in a 5% CO_2_ incubator to induce the AR. Percentage of spontaneous and A23187-induced AR following this was determined using FITC-conjugated *Pisum sativum* lectin (PSA-FITC) labelling (0.1 mg/ml; Sigma) at T0 and T90 minutes using sperm from wild-type +/+ and *DefbΔ9* −/− mice as previously described [41]. Briefly, ∼30 µl sperm suspension was smeared onto a slide, air dried then fixed and permeabilized in 100% methanol for 15 mins at room temperature. After methanol fixation, 100 ul PSA-FITC lectin (0.1 mg/ml) was added to the slide and incubated in the dark for 30 mins at room temperature. The slides were rinsed with PBS and mounded with fluorescent mounting media. One to two hundred sperm were scored and classified as “acrosome-intact” or “acrosome reacted” using epifluorescence and phase contrast microscopy at ×40 magnification.

### Zonadhesin antibody staining

Zonadhesin (ZAN) is a sperm-specific protein located in the acrosome and is critically involved in sperm-ZP adhesion. Live, motile sperm expose ZAN at the surface when cells are capacitated. Therefore ZAN exposure is an alternative indirect way of measuring sperm capacitation. ZAN was detected by incubating cells in suspension with anti-zonadhesin D3p18 domain (1 µg/ml) affinity-purified antibodies at 37°C for 30 mins as previously described [42]. After spinning cells at 500× g for 5 mins, bound antibodies were visualised with a goat anti-rabbit IgG conjugated to Alexa Fluor 594 (3 µg/ml; Molecular Probes) on cells in suspension or on cells smeared and dried on slides as for immunofluorescence on fixed cells.

### Acrosome integrity determination by Coomassie stain

Cauda sperm were isolated in modified Tyrode's medium, fixed in 4% formalin for 15 mins. Sperm were spun down (1000× g 5 mins) then washed once in 0.1 M ammonium acetate (pH 9.0) and resuspended in a final volume of approximately 50 µl. An aliquot of 20 µl was smeared on glass slide, allowed to air dry then stained for 2 mins with a solution of 0.22% Coomassie blue G250 (wt/v) in 50% methanol (v/v) and 10% acetic acid (v/v). Slides were washed 3 times in distilled water, air-dried and mounted in glycerol. Acrosome integrity of at least 150 sperm per animal was assessed by the presence or absence of an intense blue stain on the anterior sperm head (mean ± SD; n = 3 pairs).

### Statistical analysis

Results are expressed as mean± standard deviation (SD). Statistical differences between groups were tested by the Student t-test or non-parametric Mann-Whitney test as appropriate. A p-value of <0.05 was considered to be significant.

## Supporting Information

Figure S1 Gene targeting of the 9 β-defensin genes using MICER vectors. *Figure S1A: ES cell clone 199 is targeted to DNA telomeric to Defb13 and is PCR positive (upper panel) and hybridizes with internal oligo (lower panel).* A MICER clone carrying exons 1 and 2 of the *HPRT* gene, a *neo* selection cassette and the *Tyrosinase* gene and 7 Kb of homology to the genomic region downstream of *Defb13* was constructed. The targeted clone 199 (lane 17, *) was isolated with a long range PCR from vector DNA to genomic DNA outside the vector (upper panel). Southern blot using an internal oligonucleotide validated the PCR fragment (lower panel). Primers were 5′GGGAAGTCAGGTCTATTCAG for genomic sequence not in the vector and vector sequence primer was 5′CCTTTGAGTGAGCTGATACCG and internal oligo for hybridization was 5′ACCGAGCGCAGCGAGTCAG. Clones were isolated at a frequency of 1 in 203. *Figure S1B: Clone 199 was retargeted and correctly targeted clones were PCR positive.* Clone 199 was subjected to a second round of targeting to the region upstream of *Defb1* using the MICER clone MHPP423o12 (obtained from the Wellcome Trust Sanger Institute) which has 9 Kb of homology to the mouse genome and carries the *HPRT* exons 3–8 and *puromycin* selection gene. BglII digestion removed an 840 bp fragment of DNA from the genomic DNA in the vector. Correctly targeted clones were isolated at a frequency of 1 in 4 (indicated with asterisks). PCR primers and hybridization primer were vector primer 5′GAAGACAATAGCAGGCATGCTGG and primer designed to the genomic DNA removed from the vector 5′CCATTCTTATTAAATGAGTAACTC. Internal hybridization oligo (data not shown) was GGTGGGCTCTATGGGTTCTG annealed at 68°C. *Figure S1C: Following addition of cre recombinase HAT resistant clones are isolated at high frequency.* Correctly targeted clones were isolated after expansion of cre recombinase, which will create a functional *HPRT* gene allowing growth of the *HPRT* mutant E14 cells in HAT selection. Some clones produced HAT resistant clones at a frequency that was at least 10 fold lower than other clones, perhaps due to intra versus inter chromosomal recombination as described previously [16]. We isolated HAT resistant clones that were most efficient at producing colonies after cre exposure and selection. As expected these HAT resistant clones were puromycin and G418 sensitive as the plasmid sequences containing these selection cassettes were lost during the recombination. PCR of the *HPRT* gene showed sequence consistent with the expected *lox*-mediated recombination event (data not shown and Fig. 1B in main text).
(TIF)

Figure S2 Expression level of genes not in the *DefbΔ9* deletion was unaltered in the epididymis. Expression levels of Bin1b (*Spag11*) (A) and *Defb33* (B) determined by qRT-PCR were not found to be affected by the deletion of the 9 defensin gene cluster on chromosome 8 from cDNA samples prepared from the epidiymis of wild type (+/+) or *DefbΔ9* (−/−) mice. Each sample was analysed in triplicate.
(TIF)

Figure S3 Level of antimicrobial killing against *Pseudomonas aeruginosa* O1 (PAO1) by the reduced and oxidized peptides. β-defensins with the mature peptide sequences (as shown in Table 1) were purchased from Almac Sciences (Scotland) Limited in an oxidised form. They were tested for their ability to kill PAO1 in 3 hours at various concentrations of peptide. As none of the peptides had a minimum bactericidal concentration below 50 µg/ml, this high level was used to assess the effect of the non-reversible reducing agent Tris (2-carboxyethyl) phosphine (TCEP) on the killing ability of the peptides. Reduced β-defensin peptides have been shown to have additional antimicrobial activity in some cases [19]. TCEP alone had no effect on bacterial survival (data not shown). TCEP reduction resulted in an increase in bacterial killing of all the peptides except Defb15, which remained unremarkable at this concentration of peptide.
(TIF)

Figure S4 Ultrastructure of wild-type sperm exposed to calcium ionophore A23187 induces a defect in microtubule structure. TEM of wild-type cauda sperm incubated with 1 µM of calcium ionophore A23187 at T90 minutes. This treatment induces disruption of axonemal microtubules in the tails (arrowheads) of wild-type mice and this phenotype is similar to the defect observed in sperm from the *DefbΔ9* (−/−) mice (Fig. 5A). Panel below shows abnormal tail score of wild-type sperm after A23187 treatment (+A23) and the respective control in capacitation medium (+CM) without A23187 at T90 minutes time point. Following A23187 induction, 52% of the sperm show abnormal microtubule structure (101/195) compared to 3% of the control sample (3/105).
(TIF)

Table S1 Primer sequences for PCR and quantitative PCR. *Table S1A:* Primer sequences and annealing temperature for genomic Defensin gene PCR. *Table S1B:* Primer sequences and annealing temperature for amplification of cDNA. *Table S1C:* Primer sets used for quantitative PCR.
(TIF)
