# Supplementary figures and images for: Partial Deletion of Chromosome 8 β-defensin Cluster Confers Sperm Dysfunction and Infertility in Male Mice
Source: PLoS Genet. 2013 Oct 24;9(10):e1003826. doi: 10.1371/journal.pgen.1003826 (PMC3812073; doi:10.1371/journal.pgen.1003826)

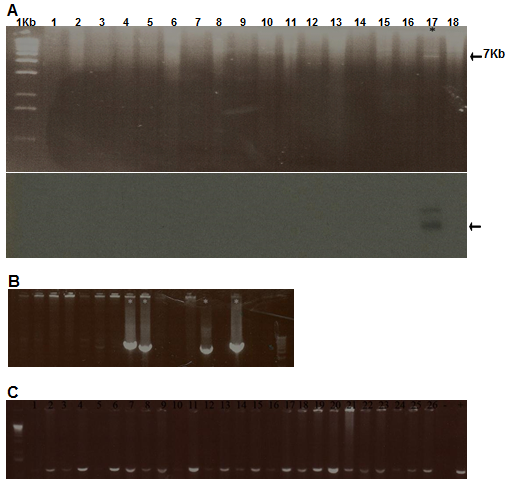

Supplement: Figure S1 — Gene targeting of the 9 β-defensin genes using MICER vectors. Figure S1A: ES cell clone 199 is targeted to DNA telomeric to Defb13 and is PCR positive (upper panel) and hybridizes with internal oligo (lower panel). A MICER clone carrying exons 1 and 2 of the HPRT gene, a neo selection cassette and the Tyrosinase gene and 7 Kb of homology to the genomic region downstream of Defb13 was constructed. The targeted clone 199 (lane 17, *) was isolated with a long range PCR from vector DNA to genomic DNA outside the vector (upper panel). Southern blot using an internal oligonucleotide validated the PCR fragment (lower panel). Primers were 5′GGGAAGTCAGGTCTATTCAG for genomic sequence not in the vector and vector sequence primer was 5′CCTTTGAGTGAGCTGATACCG and internal oligo for hybridization was 5′ACCGAGCGCAGCGAGTCAG. Clones were isolated at a frequency of 1 in 203. Figure S1B: Clone 199 was retargeted and correctly targeted clones were PCR positive. Clone 199 was subjected to a second round of targeting to the region upstream of Defb1 using the MICER clone MHPP423o12 (obtained from the Wellcome Trust Sanger Institute) which has 9 Kb of homology to the mouse genome and carries the HPRT exons 3–8 and puromycin selection gene. BglII digestion removed an 840 bp fragment of DNA from the genomic DNA in the vector. Correctly targeted clones were isolated at a frequency of 1 in 4 (indicated with asterisks). PCR primers and hybridization primer were vector primer 5′GAAGACAATAGCAGGCATGCTGG and primer designed to the genomic DNA removed from the vector 5′CCATTCTTATTAAATGAGTAACTC. Internal hybridization oligo (data not shown) was GGTGGGCTCTATGGGTTCTG annealed at 68°C. Figure S1C: Following addition of cre recombinase HAT resistant clones are isolated at high frequency. Correctly targeted clones were isolated after expansion of cre recombinase, which will create a functional HPRT gene allowing growth of the HPRT mutant E14 cells in HAT selection. Some clones produced HAT resistant clones [file pgen.1003826.s001.tif]

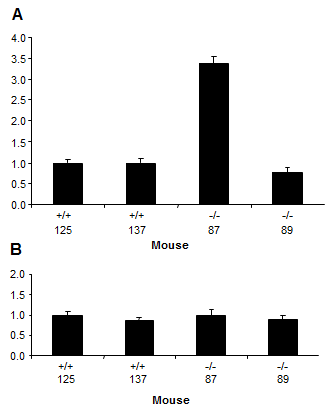

Supplement: Figure S2 — Expression level of genes not in the DefbΔ9 deletion was unaltered in the epididymis. Expression levels of Bin1b (Spag11) (A) and Defb33 (B) determined by qRT-PCR were not found to be affected by the deletion of the 9 defensin gene cluster on chromosome 8 from cDNA samples prepared from the epidiymis of wild type (+/+) or DefbΔ9 (−/−) mice. Each sample was analysed in triplicate. (TIF) [file pgen.1003826.s002.tif]

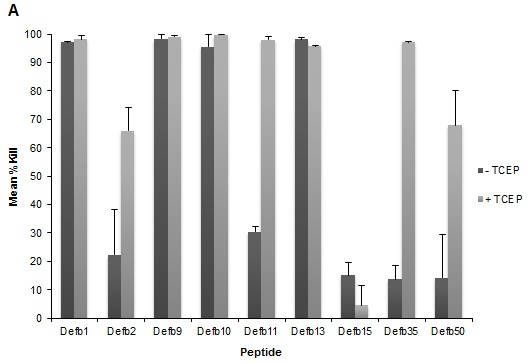

Supplement: Figure S3 — Level of antimicrobial killing against Pseudomonas aeruginosa O1 (PAO1) by the reduced and oxidized peptides. β-defensins with the mature peptide sequences (as shown in Table 1) were purchased from Almac Sciences (Scotland) Limited in an oxidised form. They were tested for their ability to kill PAO1 in 3 hours at various concentrations of peptide. As none of the peptides had a minimum bactericidal concentration below 50 µg/ml, this high level was used to assess the effect of the non-reversible reducing agent Tris (2-carboxyethyl) phosphine (TCEP) on the killing ability of the peptides. Reduced β-defensin peptides have been shown to have additional antimicrobial activity in some cases [19]. TCEP alone had no effect on bacterial survival (data not shown). TCEP reduction resulted in an increase in bacterial killing of all the peptides except Defb15, which remained unremarkable at this concentration of peptide. (TIF) [file pgen.1003826.s003.tif]

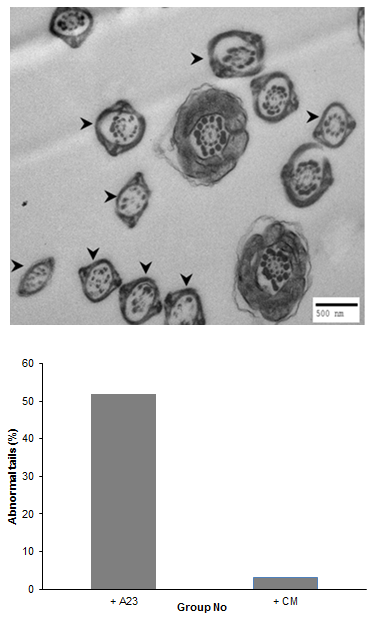

Supplement: Figure S4 — Ultrastructure of wild-type sperm exposed to calcium ionophore A23187 induces a defect in microtubule structure. TEM of wild-type cauda sperm incubated with 1 µM of calcium ionophore A23187 at T90 minutes. This treatment induces disruption of axonemal microtubules in the tails (arrowheads) of wild-type mice and this phenotype is similar to the defect observed in sperm from the DefbΔ9 (−/−) mice (Fig. 5A). Panel below shows abnormal tail score of wild-type sperm after A23187 treatment (+A23) and the respective control in capacitation medium (+CM) without A23187 at T90 minutes time point. Following A23187 induction, 52% of the sperm show abnormal microtubule structure (101/195) compared to 3% of the control sample (3/105). (TIF) [file pgen.1003826.s004.tif]

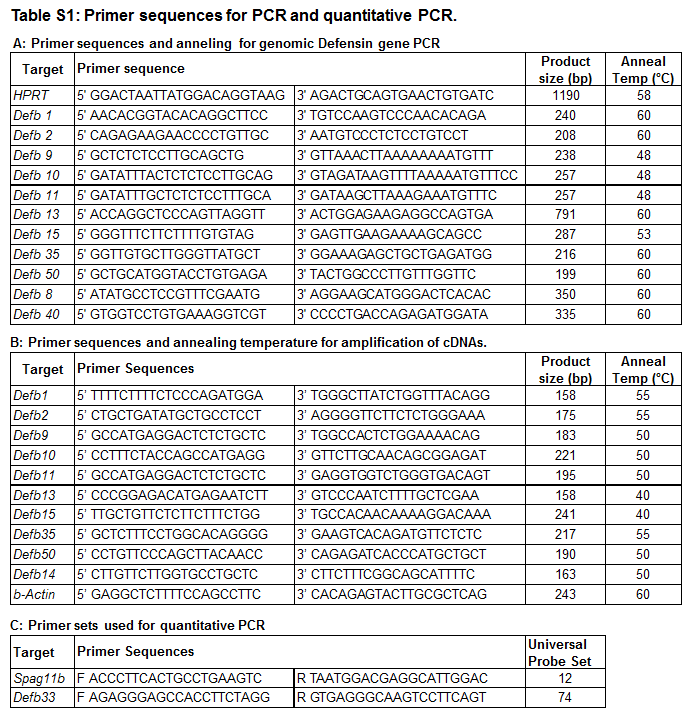

Supplement: Table S1 — Primer sequences for PCR and quantitative PCR. Table S1A: Primer sequences and annealing temperature for genomic Defensin gene PCR. Table S1B: Primer sequences and annealing temperature for amplification of cDNA. Table S1C: Primer sets used for quantitative PCR. (TIF) [file pgen.1003826.s005.tif]
